# Salvage of post surgical scaphoid non-union

**DOI:** 10.1186/1753-6561-9-S3-A41

**Published:** 2015-05-19

**Authors:** Juichi Tanaka

**Affiliations:** 1 Department of Orthopaedic Surgery, Hyogo College of Medicine, 663-8131, Japan

## Introduction

Acceptable results of scaphoid fracture have been reported associated with development of surgical procedures and improvement of fixation screws. However, some scaphoid fractures result in nonunion after surgery. Nonunion of scaphoid fracture causes osteoarthritis of the wrist. Therefore, it is important to achieve union. In the present study, we retrospectively analyzed the salvage operation of fracture nonunion of the scaphoid, which had been treated surgically but did not unite.

## Method

In the salvage operation, we removed the screw which had been used in the previous surgery and insert the cortical bone peg in the screw hole. The cancellous bone taken from the iliac crest was grafted at the nonunion site and fracture was fixed with a headless double threaded screw, such as DTJ screw or Herbert screw.

We treated 32 patients (male 28, female 4) and the average age at the salvage operation was 26.3 (range from 16 to 45). The diagnosis at the previous surgery was acute scaphoid fracture in 14 patients and nonunion of the scaphoid in 11 patients. All patients had been treated surgically, however, union was not obtained and the salvage operation was needed. The average duration from the previoussurgery to the salvage operation is 34.2 months.

## Results

Union of scaphoid fracture was confirmed in 31 patients after the first salvage operation. One patient, who did not achieve union by the first salvage surgery, was treated with the second salvage operation and union was obtained.

The average dorsal flexion of the wrist joint was 57 degree before the salvage operation and 70 degree after the salvage operation. The average palmar flexion of the wrist joint was 65 degree before the salvage operation and 70 degree after the salvage operation. The average grip strength was 36 kg before the salvage surgery and 44kg after the salvage surgery.

## Summary points

The procedures of the salvage operation to fracture nonunion of the scaphoid were a removal of the screw, an insertion of a cortical bone peg in the screw hole, an iliac bone graft to the fracture site, and an internal fixation with headless screw.

This procedure is effective as a salvage operation to fracture nonunion of the scaphoid.

